# A study on the explicit self-injurious behaviors of adolescent patients with depression, their implicit self-injurious attitudes, and their relationship with self-esteem levels

**DOI:** 10.3389/fpubh.2026.1876805

**Published:** 2026-07-15

**Authors:** Chaona Shang, Fang Yan, Yuhua Qiu, Shuqin Zhao, Junlei Zhang

**Affiliations:** 1 Department of Child and Adolescent Psychiatry, The Second Affiliated Hospital of Henan Medical University, Xinxiang, China; 2 Nursing Department, The Second Affiliated Hospital of Henan Medical University, Xinxiang, China

**Keywords:** adolescent, depression, implicit association test, non-suicidal self-injury, self-esteem

## Abstract

**Objective:**

Non-suicidal self-injury behaviors are relatively common among adolescents with depression, seriously endangering their physical and mental health as well as the prognosis of the disease. Currently, the relationship between explicit self-injury behaviors, implicit self-injury attitudes and self-esteem levels of adolescents with depression is not clear. Therefore, this study systematically analyzed the correlation between explicit self-injury behaviors, implicit self-injury attitudes and self-esteem levels of adolescents with depression, aiming to provide theoretical basis for reducing self-injury behaviors and improving their self-esteem levels.

**Methods:**

A cross-sectional study was conducted from April 2025 to March 2026 at a mental health specialty hospital in Xinxiang City, Henan Province, China. Using the convenience sampling method, 116 adolescent patients with depression were selected to measure the Non-Suicidal Self-Injury Behavior Questionnaire, the Self-Esteem Level Scale, and to explore the patients' implicit attitudes toward self-injury through the Implicit Association Test.

**Results:**

The score of explicit self-injury behavior in adolescent patients with depression was (25.66 ± 12.11); Correlation analysis showed that the explicit self-injury behavior of the patients was significantly negatively correlated with their self-esteem level (*P* < 0.05). The effect value of the implicit self-injury attitude of the patients was (0.26 ± 1.06), which was different from 0 and the difference was statistically significant; this suggested that the patients had a significant implicit tendency toward self-injury; there was no significant correlation between the implicit self-injury attitude and the explicit self-injury behavior, or the self-esteem level (*P* > 0.05).

**Conclusion:**

The explicit self-injury behaviors of adolescent patients with depression are at a medium-high level, and the implicit self-injury attitudes are significant and have an independent association effect. When conducting intervention for adolescent patients with depression in clinical settings, it is necessary to abandon the single explicit behavior intervention model and take into account the patient's implicit cognitive characteristics and self-esteem level. A comprehensive intervention system that integrates both internal and external aspects should be constructed to effectively reduce the risk of self-harm behaviors, enhance the patient's self-esteem level and overall mental health level.

## 1 Introduction

Self-esteem refers to the positive perception and evaluation of one's own value by an individual, which is an important internal driving force that promotes the realization of one's own value and is closely related to physical and mental health (1). High levels of self-esteem can help adolescent patients with depression cope with stressful events or traumatic experiences and reduce the negative psychological and behavioral impacts brought about by negative life events (2). Self-esteem is closely related to non-suicidal self-injury behavior. Lei et al. (3) found that the self-esteem level of adolescents with self-injury was significantly lower than that of the healthy control group. Yenen et al. (4) suggested that the self-esteem level of adolescents is significantly negatively correlated with self-injury behavior, and self-esteem plays a mediating or regulatory role between self-injury and other variables. The self-injury integration theory holds that self-esteem is a protective factor in the motivational pre-stage, and the self-esteem level can regulate the motivational process of self-injury impulses triggered by stress triggers (5).

Non-suicidal self-injury (NSSI) refers to the repetitive and deliberate self-injury of one's body tissues without the intention of suicide, and is an act that is not accepted or recognized by society. It includes cutting, hitting, hindering wound healing, etc. (6). NSSI is highly prevalent among the youth population. The global prevalence of NSSI among teenagers is 17.2%, and in China, it is as high as 24.7% (7). The dual attitude model clearly states that individuals have two independent cognitive systems for a certain behavior or object: an implicit automatic attitude and an explicit controllability attitude. The implicit attitude stems from the long-term, subconscious accumulation of individual experiences and belongs to unconscious, automatic, and difficult to deliberately regulate cognitive connections, with higher stability. The explicit attitude is an individual's conscious, subjective modifiable, and socially approved effect-influenced controllable cognitive expression. The two can exist independently and separately, or interact with each other (8). According to the dual attitude model, an individual's attitude toward self-injury can be divided into two levels: explicit and implicit. That is, when an individual consciously denies having thoughts of self-injury, their implicit attitude can still be unconsciously activated and become the internal driving force for self-injury behavior in specific situations (9). Therefore, relying solely on explicit measurement tools to assess the frequency and severity of NSSI has limitations. When an individual conceals the truth due to feelings of stigma or avoidance of treatment, the explicit report is difficult to accurately reflect their true risk of self-injury.

The Self-Injury Implicit Association Test (SI-IAT) is based on the dual-attitude model and uses reaction time indicators to detect automatic cognitive associations between individuals and self-injury behaviors. It effectively avoids the reporting biases of explicit assessments and improves traditional measurement methods (10). Studies abroad have shown that the SI-IAT can reveal the hidden cognitive tendencies of individuals before they express suicidal intentions and behaviors, and can predict suicidal attempts within 6 months (11). Domestic investigations have found that patients with schizophrenia have a higher implicit attitude toward self-injury, and the SI-IAT can effectively screen and identify suicide risks (12). However, research combining NSSI with implicit attitudes in China is still in its infancy, lacking systematic introduction of the dual-attitude model into clinical studies of adolescent depression patients, and the predictive and specific associations between implicit attitudes toward self-injury and the risk of self-injury suicide still lack sufficient evidence.

Self-esteem, as the core evaluation system of an individual's self-worth, the level of self-esteem can significantly predict self-injury behaviors. Exploring the correlation between implicit self-injury attitudes and the level of self-esteem holds significant clinical importance for adolescent patients with depression (13). Previous studies have mostly focused on the relationship between explicit self-injury behaviors and the level of self-esteem, while rarely paying attention to the relationship between implicit self-injury attitudes and explicit self-injury behaviors, as well as the level of self-esteem. Therefore, in the framework of the dual attitude model and the self-injury integration theory, this study focuses on the adolescent depression patient group to analyze the relationship among explicit self-injury behaviors, implicit self-injury attitudes, and the level of self-esteem. Based on this, the following hypotheses are proposed: H1: The level of self-esteem in adolescent depression patients is significantly negatively correlated with explicit self-injury behaviors, that is, the lower the level of self-esteem, the higher the frequency and severity of the individual's explicit NSSI behaviors. H2: The implicit self-injury attitude of adolescent depression patients has a significant positive correlation with explicit NSSI behaviors. The stronger the implicit self-injury cognitive tendency, the more prominent the explicit self-injury behavior. H3: There is no significant correlation between the implicit self-injury attitude of adolescent depression patients and the level of self-esteem. The implicit self-injury attitude is independent of the explicit self-esteem system, and there is an attitude separation feature between the two.

## 2 Participants and methods

### 2.1 Participants

A total of 116 adolescent inpatients with depression were recruited from a specialized psychiatric hospital in Henan Province using the convenience sampling method. **Inclusion criteria were as follows:** Met the diagnostic criteria for non-suicidal self-injury in the Diagnostic and Statistical Manual of Mental Disorders, 5th Edition (DSM-5) published by the American Psychiatric Association (14); Met the diagnostic criteria for depressive disorder in the ICD-10 Classification of Mental and Behavioral Disorders: Clinical Descriptions and Diagnostic Guidelines (15); Aged 11–18 years; Able to independently complete relevant questionnaires and the Implicit Association Test (IAT);? Hamilton Depression Rating Scale score ≥ 7 and Hamilton Anxiety Rating Scale score ≥ 14. **Exclusion criteria were as follows:** Presence of severe physical diseases including cardiac, hepatic, or renal insufficiency; Comorbidity with other psychiatric disorders. The sample size was calculated using the G^*^Power software for sample size estimation. The two-tailed test was set with α = 0.05 and the test power 1 - β = 0.8. A medium effect size *r* = 0.30 was selected. Considering a data loss rate of 10%−15% in the Implicit Association Test, a total of 116 research subjects were finally included.

All participants voluntarily enrolled in this study, and written informed consent was obtained from both patients and their guardians. This study was approved by the Medical Ethics Committee of the Second Affiliated Hospital of Henan University of Chinese Medicine (Ethics No.: XYEFYLL-2023-45).

### 2.2 Methods

#### 2.2.1 Measurement tools

##### 2.2.1.1 General information questionnaire

A self-designed questionnaire was developed based on relevant domestic and international literature, including gender, age, only-child status, place of residence, and family structure.

##### 2.2.1.2 Explicit self-injury behavior measurement tool

The Non-Suicidal Self-Injury Behavior Questionnaire developed by Wan Yuhui (16) was used to assess whether individuals had engaged in non-suicidal self-injury in the past year and its frequency. The questionnaire consists of 12 items with a 5-point Likert scale. Higher scores indicate more severe self-injurious behavior. The Cronbach's α coefficient of this scale in adolescent patients with depression was 0.921, indicating good reliability and validity.

##### 2.2.1.3 Self-esteem scale

The Self-Esteem Scale was developed by Rosenberg (17) in 1965 to evaluate individual self-worth and self-competence. It contains 10 items using a 4-point Likert scale, among which items 3, 5, 8, 9, and 10 are reverse-scored. Higher total scores represent higher self-esteem levels. The Cronbach's α coefficient of this scale was 0.78, showing good reliability and validity.

#### 2.2.2 Implicit measurement tool

In this study, concept words (self-related words/other-related words) and attribute words (life-related words/self-injury words) suitable for adolescent patients with depression were selected through a pre-experiment, and the Self-Injury Implicit Association Test procedure was compiled using E-Prime 3.0 software (Psychology Software Tools, Inc., Pittsburgh, PA, United States).

##### 2.2.2.1 Experimental materials

Based on literature review and the Modern Chinese Dictionary, 20 concept words (self-related words and other-related words) and 20 attribute words (life-related words and self-injury words) were screened. Twenty postgraduate students majoring in psychology rated these words using a 5-point Likert scale, with higher scores indicating higher matching degree. The final target words were determined and are shown in Table 1.

**Table 1:** Vocabulary for implicit association test.

Conceptual terms	Attributive terms
Self-referential terms: myself, we, I, self, I	Self-injury terms: scratching, self-injury, cutting, banging my head against the wall, picking at scabs
Other-referential terms: others, his, they, others, he	Survival terms: laughter, survival, being alive, life, growing up

##### 2.2.2.2 Implicit test procedure

The implicit test procedure was programmed using E-Prime 3.0. In this study, participants' implicit attitudes toward survival and self-injury were measured based on their reaction times to survival/self and self-injury/self. The task included 10 attribute words (self-injury-related words, survival-related words) and 10 concept words (self-related words, non-self-related words). Following Nock's (18) experimental procedure, participants were required to perform attribute discrimination on self-injury-related and survival-related words, and concept discrimination on self-related and non-self-related words. The target words were paired with attribute words to form “compatible tasks” and “incompatible tasks.” The difference between the compatible and incompatible tasks serves as an indicator of the intensity of implicit attitudes. This experiment consists of 7 steps, with 20 trials to be completed in each step; the entire process takes approximately 15 min. Detailed procedural steps are provided in Table 2.

**Table 2:** Procedures of the implicit self-injury association test.

Step	Name	Trial	Reaction key	
			*F*	*J*
1	Practice	20	Alive	Self-injury
2	Practice	20	Self	Non-self
3	Practice	20	Alive/self	Self-injury/non-self
4	Test	20	Alive/self	Self-injury/non-self
5	Practice	20	Self-injury	Alive
6	Practice	20	Self-injury/self	Alive/non-self
7	Test	20	Self-injury/non-self	Alive/non-self

### 2.3 Statistical methods

Data were extracted and organized using Excel, and statistical analysis was performed using SPSS 25.0. Demographic characteristics of the participants were described using frequencies and percentages. Since the scores for explicit self-injury behaviors and self-esteem did not follow a normal distribution, Spearman's correlation analysis was used. Data from the implicit association test for self-injury were processed as follows: (1) Data with reaction times greater than 10,000 ms or less than 300 ms were excluded, as were data with error rates exceeding 20%; (2) Reaction times for erroneous trials were corrected by substituting them with a value of 600 ms; (3) The difference between the average reaction times for each participant under compatible and incompatible conditions was calculated, yielding the D-score for the Implicit Self-Injury Association Test. A negative D value indicates that the subject's reaction time in the congruent task is slower than in the incongruent task, while a positive D value indicates the opposite. In this study, the D value is used as the metric for the Implicit Self-Injury Association Test, and Spearman's correlation analysis is employed to examine the relationships between subjects' implicit self-injury attitudes and their explicit self-injury behaviors and self-esteem levels. The significance level is set at α = 0.05.

## 3 Results

### 3.1 General characteristics of participants

A total of 120 questionnaires were collected, with 4 invalid responses, resulting in a valid response rate of 96.6%. The mean age of all participants was (14.18 ± 2.16); among them, 45 were male (38.79%), 71 were female (61.21%); 36 (31%) lived in rural areas, and 80 (69%) lived in urban areas; 107 (92.2%) were not only children, and 19 (16.4%) came from single-parent or blended families.

### 3.2 Self-esteem levels, scores on explicit self-injurious behavior, and their correlations among adolescents with depression

The self-esteem score of adolescent patients with depression was (24.03 ± 6.59), and the score of explicit self-injury behavior was (25.66 ± 12.11). The correlation analysis showed that self-esteem level was significantly negatively correlated with explicit self-injury behavior (*r* = −0.710, *P* < 0.05). After controlling for variables such as age and gender, the partial correlation analysis still showed statistical significance, indicating that self-esteem is independently associated with explicit self-injury behavior.

### 3.3 Analysis of differences in reaction times between compatible and incompatible trials

All participants in this study underwent the Implicit Association Test for self-injury. The mean score for the compatible condition was 1,400.260 ± 320.590, while the mean score for the incompatible condition was 1,488.331 ± 342.632. Compared to the incompatible condition, participants' reaction times on the Implicit Association Test for self-injury were shorter in the compatible condition (*t* = 2.576, *P* < 0.05), indicating that participants have formed stronger automatic associations between their self-concept and self-injury-related concepts at the unconscious level, demonstrating an implicit preference for or implicit identification with self-injury.

### 3.4 Implicit effect size of participants' implicit self-injury attitudes

The D value of the implicit self-injury attitude of the 116 participants was (0.26 ± 1.06), and compared with 0, the difference was statistically significant (*t* = 2.581, *P* < 0.05), indicating that the implicit self-injury attitude of the participants was relatively significant.

### 3.5 Correlations between participants' implicit self-injury attitudes and explicit self-injury behaviors and self-esteem levels

The D-scores from the implicit self-injury association test showed no significant correlations with scores on explicit self-injury behaviors or self-esteem levels (*P* > 0.05), as shown in Table 3.

**Table 3:** Correlations among implicit self-injury attitudes, explicit self-injury behavior, and self-esteem level in participants (*n* = 116, *r*).

Item	*D*-value	*P*-value
Explicit self-injury behaviors	−0.049	0.620
Self-esteem level	0.051	0.604

## 4 Discussion

### 4.1 Analysis of the current status of explicit self-injurious behavior, implicit attitudes toward self-injury, and self-esteem levels Among adolescents with depression

The scores for explicit self-injury behaviors among adolescents with depression (25.66 ± 12.11) were at a medium-to-high level. This is consistent with the research results of Yuan et al. (19), but there are significant differences in the survey subjects and age. In this study, the survey subjects included all hospitalized adolescent patients with depression who were constantly in a high-pressure medical care environment. Their negative emotions and impulsive behaviors were higher than those in the outpatient population. This is also related to the fact that the emotional regulation ability of adolescents is not yet mature, and depressive emotions can be easily expressed through explicit self-injury behaviors; at the same time, the depressive state often accompanies negative cognitive biases, making individuals more likely to adopt self-injury coping strategies in stressful situations, and thus presenting a higher level of explicit self-injury behaviors. Therefore, nursing staff should prioritize the early screening and dynamic assessment of non-suicidal self-injury behaviors in adolescents with depression. They should strengthen training in emotional regulation, impulse control, and stress coping skills and guide adolescents toward healthy and effective ways of expressing emotions. At the same time, by collaborating with families, schools, and multidisciplinary teams, a systematic and continuous psychological nursing intervention system should be established to reduce the risk of self-injury and safeguard the physical and mental health of adolescents.

The results of the implicit association test on self-injury indicate that self-injury is not merely an overt coping behavior but rather a deep-seated, automatic, and often unnoticed cognitive tendency whereby individuals habitually and automatically associate the “self” with “self-injury.” This is similar to the research results of Franklin JC et al. (20) and others regarding self-injury among adolescents in European and American communities. However, there are differences in population background such as region, culture, and diagnostic status. Compared with the community groups, the patients in this study have more typical psychopathological symptoms, more fixed pathological implicit cognitive connections, and the degree of depression will further strengthen the unconscious connection between “self” and “self-injury,” thereby increasing the risk of self-injury. The self-injury behaviors of adolescent patients with depression have formed a stable implicit cognitive pattern. Their identification and inclination toward self-injury do not merely stem from a momentary emotional impulse but have already formed pathological cognitive connections at the unconscious level. This also indicates that relying solely on external observation and self-assessment is insufficient for a comprehensive identification of the risk of self-injury. The self-injury behaviors of adolescent patients with depression have formed a stable implicit cognitive pattern. Their identification and inclination toward self-injury do not merely stem from a momentary emotional impulse but have already formed pathological cognitive connections at the unconscious level. This also indicates that relying solely on external observation and self-assessment is insufficient for a comprehensive identification of the risk of self-injury. Notably, using the time at which participants completed the implicit association test on self-injury as a reference point, previous research has shown (21) that the degree of identification with implicit attitudes toward self-injury is stronger when self-injury behaviors occurred more recently or were more severe. Some scholars have also suggested (22) that an individual's attitude toward a specific behavior is not static but rather possesses a certain degree of plasticity; consequently, attitudes toward non-suicidal self-injury are also subject to dynamic change. This implies that frequent self-injurious behaviors triggered by acute stress will decrease as the stressor weakens or is eliminated. and the individual's implicit attitude toward NSSI will change accordingly (23). Therefore, it is recommended to combine assessments of explicit behaviors with implicit cognitive evaluations, utilizing tools such as the Implicit Association Test for Self-Injury to improve the accuracy of self-injury risk identification. At the same time, dynamic, time-series assessments should be implemented, with a particular focus on adolescents with depression who have recently engaged in self-injury or are in an acute stress phase. In response to the pathological implicit cognitive associations that have been formed, implicit cognitive bias modification training (ICBT) is introduced. Through repetitive semantic association tasks and deviation attention training, the automatic association between “self” and “self-injury” is gradually broken, and unconscious cognitive biases are reshaped (24). Additionally, it can be combined with mindfulness acceptance therapy to reduce individuals' avoidance tendencies toward negative emotions and decrease impulsive self-injury behaviors driven by implicit cognition.

This study found that the self-esteem score of adolescent patients with depression was (24.03 ± 6.59), indicating a low level of self-esteem overall. This was lower than the survey conducted by Niu et al. (25) among students in school. It may be related to multiple factors such as impaired social function, the torment of the disease, and the long-term separation from campus life of hospitalized adolescent patients with depression. Research suggests (26) that low self-esteem is a significant risk factor for the persistence and exacerbation of depressive symptoms; it can further intensify depressive mood by reinforcing negative self-evaluations and reducing emotional regulation abilities and coping resources. Conversely, high self-esteem helps individuals develop positive self-perceptions, enhances emotional regulation abilities and psychological resilience, reduces the occurrence of negative emotions, boosts self-confidence, and promotes the alleviation of depressive symptoms and the restoration of social functioning (27). Therefore, it is recommended that nursing managers prioritize the assessment of self-esteem levels in adolescents with depression. To address patients' negative cognitive biases, they should develop personalized psychological interventions—such as cognitive-behavioral therapy and narrative nursing—to help patients establish a positive self-image.

### 4.2 Analysis of the correlation between self-esteem levels and explicit self-injury behaviors in adolescents with depression

This conclusion is consistent with the findings of Halicka et al. (28) and others. Possible reasons include: (1) Adolescents with low self-esteem are more prone to negative cognitive patterns such as catastrophizing and self-blame, making it difficult for them to alleviate distress through adaptive strategies; self-injury thus becomes an extreme means of expressing inner despair. (2) Adolescents with low self-esteem lack effective coping mechanisms when facing external pressures and interpersonal conflicts, leading them to use self-injury as a means of emotional release. (3) Adolescents with low self-esteem often exhibit insecure attachment patterns; they are prone to being neglected and rejected in family and peer relationships, making it difficult for them to obtain adequate social support and emotional comfort. Consequently, when experiencing negative life events, they are more likely to resort to self-injury to gain attention from others or achieve a temporary sense of psychological balance. Tian et al. (29) has found that high self-esteem serves as a protective factor against self-injury behaviors in adolescents. Individuals with high self-esteem hold more positive evaluations and perceptions of their self-worth and are more confident in facing difficulties and challenges; this positive self-perception helps them adopt constructive coping strategies in response to stress. Therefore, it is recommended that clinicians prioritize enhancing self-esteem as a key target for preventing and reducing explicit self-injury behaviors. Cognitive-behavioral therapy can be used to correct patients' negative cognitions and establish positive self-perceptions and rational attribution styles. Additionally, group counseling focused on self-esteem should be implemented to enhance self-worth through group activities and self-exploration. It is also important to focus on building a family support system, improving patients' insecure attachment patterns. and guiding family members to provide positive attention and emotional support while reducing neglect and rejection, thereby lowering the risk of self-injury.

### 4.3 Analysis of the correlation between implicit self-injury attitudes, self-esteem levels, and explicit self-injury behaviors among adolescents with depression

This study found that the correlation between the implicit self-injury attitude of adolescent patients with depression and their self-esteem level was not significant. This suggests that the implicit self-injury attitude is an automatic and unconscious cognitive tendency and may not be able to independently influence an individual's conscious perception of their self-esteem level. Currently, self-esteem is typically measured through self-report methods, which are susceptible to external factors such as the social desirability effect and impression management, leading to idealized results. Research has shown (30) that self-esteem also possesses an “implicit” dimension: the lower the level of implicit self-esteem, the higher the risk of suicidal ideation. This suggests that future research should continue to explore implicit self-injury attitudes and self-esteem levels from both implicit and explicit perspectives. One approach could be to incorporate implicit self-injury attitudes into tests of implicit self-esteem to ensure the stability of research findings. Therefore, it is recommended that clinicians optimize their assessment systems by establishing a dual “implicit + explicit” framework to enhance the accuracy of risk assessments for high-risk populations. At the same time, the results of this study suggest that there is no significant correlation between implicit self-injury attitudes and explicit self-injury behaviors among adolescents with depression, indicating that the two are independent of one another. Some scholars have proposed that, according to the dual-attitude model (31), implicit attitudes represent automatic, unconscious evaluations, while explicit self-injury behaviors represent controlled, conscious evaluations; the two can exist independently and exert different influences on behavior in different contexts. However, other scholars (32) argue that implicit attitudes and overt behaviors are not entirely separate but share certain cognitive representations. When self-injury is highly correlated with the self, the two exhibit strong consistency. This suggests that while clinicians work to reduce the level of self-injury attitudes in adolescents with depression, they should focus on both implicit attitudes and explicit behaviors, take individual differences into account, and implement targeted intervention measures.

## 5 Conclusion

This study found that adolescents with depression exhibit moderate-to-high levels of explicit self-injury behaviors. The implicit self-injury attitude is significant and has an independent association effect. Future interventions for adolescents with depression should comprehensively address both implicit attitudes and explicit behaviors to reduce self-injury and enhance self-esteem. This study also has some limitations: (1) This study is cross-sectional in nature; the results only indicate the relationships among variables at a single point in time. Future longitudinal studies could be conducted to further clarify the trajectories and dynamic associations among explicit behaviors, implicit self-injury attitudes, and self-esteem levels in adolescents with depression. (2) The correlation analysis in this study cannot establish causal relationships among variables; future studies could incorporate more influencing factors. (3) The sample source was limited and the sample size was small; future studies should consider expanding both the sources and size of the sample.

## Data Availability

The original contributions presented in the study are included in the article/supplementary material, further inquiries can be directed to the corresponding author.
